# A single-graft technique: Integrating Y-incision aortic annular enlargement with ascending aortic replacement

**DOI:** 10.1016/j.xjtc.2025.07.024

**Published:** 2025-08-11

**Authors:** Xusheng Zhang, Fusheng Zhang, Zhenqiang Xu, Gang Zhang

**Affiliations:** a Department of Cardiovascular Surgery, Shandong Provincial Hospital Affiliated to Shandong First Medical University, Jinan, Shandong, China; b Department of Neurology, Shandong Provincial Hospital Affiliated to Shandong First Medical University, Jinan, Shandong, China

**Figure:**
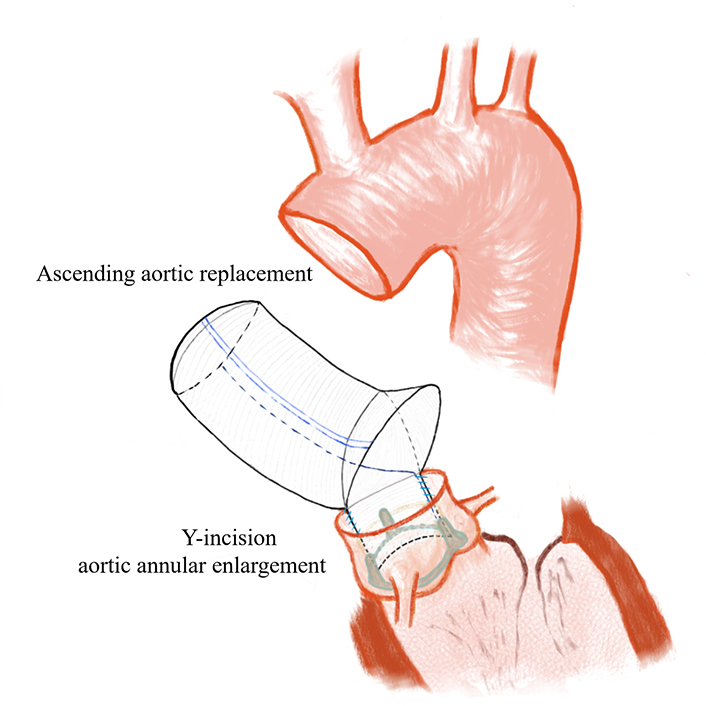
Illustration of surgical procedure for a single-graft technique.

**Central Message**

A Dacron graft integrating Y-incision aortic annular enlargement with ascending aortic replacement reduces the steps of surgical procedures, ensuring safety and effectiveness.

Dr Bo Yang's Y-incision aortic annular enlargement technique is an effective method that can safely and effectively enlarge the aortic annulus by 2 to 3 valve sizes.^1^ If an ascending aortic replacement is also needed, the artificial vessel should be anastomosed to the expanded aortic root, which will leave one suture line at the posterior wall of the root, carrying a risk of bleeding. We modified this procedure using a segment of Dacron tube graft and achieved good results.

A 54-year-old female patient (height: 160 cm; body surface area: 1.73 m^2^) presented with exercise-induced chest tightness and transient amaurosis. Echocardiography revealed a calcified bicuspid aortic valve (type 0) with severe stenosis, ie, an aortic annulus diameter of 2.0 cm and a mean pressure gradient of 74 mm Hg. The ascending aorta demonstrated a maximal internal diameter of 4.9 cm. Left ventricular hypertrophy was noted with preserved ejection fraction (left ventricular ejection fraction 58%).

## Techniques

**Figure 1:**
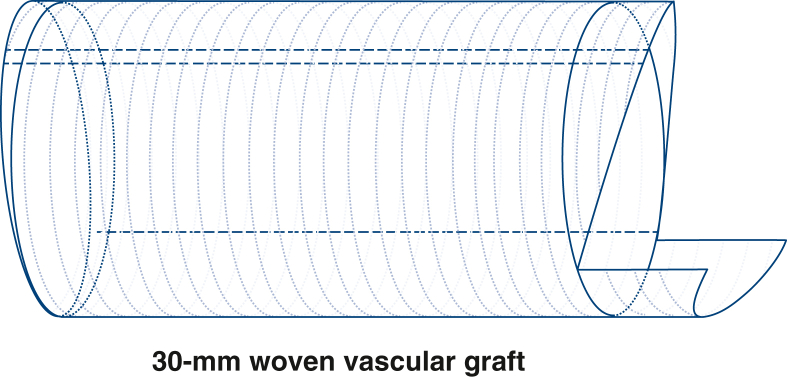
Details of artificial vessel trimming methods are shown.

Video 1 demonstrates the procedure. The aorta was sectioned transversely approximately 1 cm above the sinotubular junction, and then the severely calcified aortic valve was removed. A Y-incision was created from the postjunction parallel to the aortic annulus, undermining the aortic annuli to their respective nadirs by partial division of the left and right fibrous trigones.^1^ The end of a 30-mm Hemashield Platinum Double Velour Vascular Graft (MAQUET Cardiovascular LLC) was measured to 3 cm to form a long edge, and 2 short edges of approximately 2.5 cm were cut longitudinally separately to create a rectangular patch remaining attached at its base instead of complete transection (Figure 1). The rest of the vascular leaflet was inverted into the lumen, ensuring full exposure of the rectangular patch. A 0.5-cm high arc was excised from the long side of the rectangular patch.^2^ The patch was sewn to the aortomitral curtain from left to right fibrous trigone, following the 2 sides of the triangular-shaped aortomitral curtain with a running 4-0 PROLENE suture (Ethicon).

**Figure 2:**
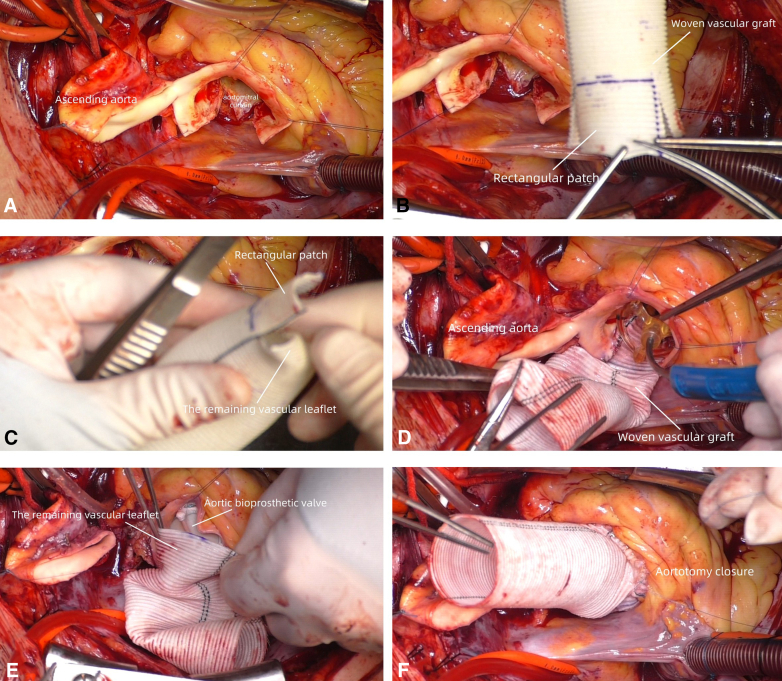
The entire process of combining the Y-incision aortic annular enlargement with ascending aortic replacement. A, Make a Y-shaped incision by posterior dissection, widely opening the aortic root. B, Trim the proximal end of the graft into a rectangular patch (connecting the base part) and make an arc modification. C, Fold the remaining part into the lumen for later use. D, Suture the rectangular patch onto the Y-shaped incision to complete Y-incision aortic annular enlargement. E, Unfold the remaining part, create a curved patch, and match it with the aortic remnant. F, Complete the closure of the anterior wall of the proximal aorta.

**Figure 3:**
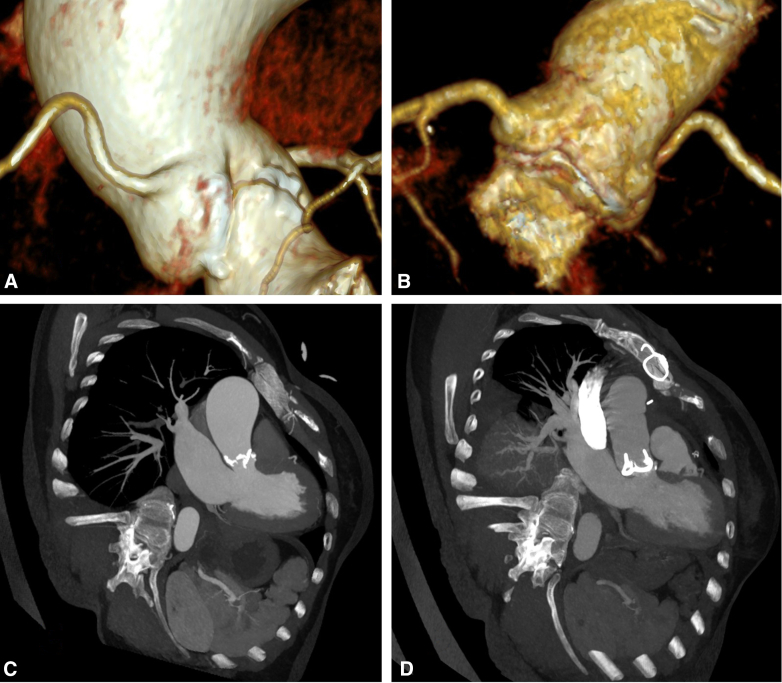
A and C, Indicate preoperative aortic valve stenosis and dilation of the ascending aorta. B and D, Show that postoperative blood flow exiting from the left ventricular outflow tract, passing through the valve, and reaching the ascending aorta is unobstructed, with no significant stenosis.

After the completion of root enlargement, a 25-mm Magna Ease pericardial bioprosthetic valve (Edwards Lifesciences) was implanted. The residual vascular leaflet within the prosthetic vessel was everted and trimmed into a curved shape toward the proximal end. The prosthetic vessel was sutured to the proximal aortic stump using 4-0 PROLENE suture. The distal vessel end was anastomosed similarly (Figure 2). Postoperative echocardiography demonstrated fixed valve position with well-opened state, revealing an aortic valve mean gradient of 9 mm Hg. Postoperative computed tomography angiography showed that blood flow was unobstructed (Figure 3). The annulus was oriented perpendicular to blood flow direction, and the positions of the coronary arteries were normal. The patient was discharged without complications. Written informed consent was provided by the patient for publication; institutional review board approval was not required.

## Discussion

Although the Wheat procedure can treat bicuspid aortic valve combined ascending aortic aneurysm, it does not address the issue of a small annulus. Moreover, the biological valves' effective orifice diameter is smaller than the labeled size, which may cause relative stenosis and patient−prosthesis mismatch.^3^^,^^4^ To overcome these limitations, previous approaches involved cutting a Dacron graft to create a separate rectangular patch for Y-incision aortic annular enlargement, analogous to the use of a Hemashield patch. However, when concomitant ascending aortic replacement is required, graft-to-graft anastomosis is performed on the aortic root posterior wall—a hidden site with difficult hemostasis. In comparison, our method eliminates the need for suturing the posterior wall, thereby maintaining its continuity. This avoids both the need for additional patch materials and reduces the risk of bleeding. Because sufficiently larger Dacron grafts were unavailable in our center, we designed a curved patch to make the aortic root smoother. If a more suitable graft is selected after the annulus is enlarged, a curved patch is not needed. In our subsequent work, we found that standardizing details can facilitate the implantation of larger valves.^4^ Furthermore, the use of larger Dacron graft and larger valves will improve hemodynamics at the aortic root and provide more anatomical space for future valve-in-valve transcatheter aortic valve implantation.^5^

## Conclusions

A single-graft reduces the steps of surgical procedures, ensuring safety and effectiveness, and can provide reference ideas for multi-region replacement surgeries.

## Conflict of Interest Statement

The authors reported no conflicts of interest.

The *Journal* policy requires editors and reviewers to disclose conflicts of interest and to decline handling or reviewing manuscripts for which they may have a conflict of interest. The editors and reviewers of this article have no conflicts of interest.

## Supplementary Data

Video 1 A novel procedure that combines Y-incision aortic root enlargement with ascending aorta replacement. Video available at: https://www.jtcvs.org/article/S2666-2507(25)00339-6/fulltext.
