# Supplementary figures and images for: A single-graft technique: Integrating Y-incision aortic annular enlargement with ascending aortic replacement
Source: JTCVS Tech. 2025 Aug 11;33:27–30. doi: 10.1016/j.xjtc.2025.07.024 (PMC12529695; doi:10.1016/j.xjtc.2025.07.024)

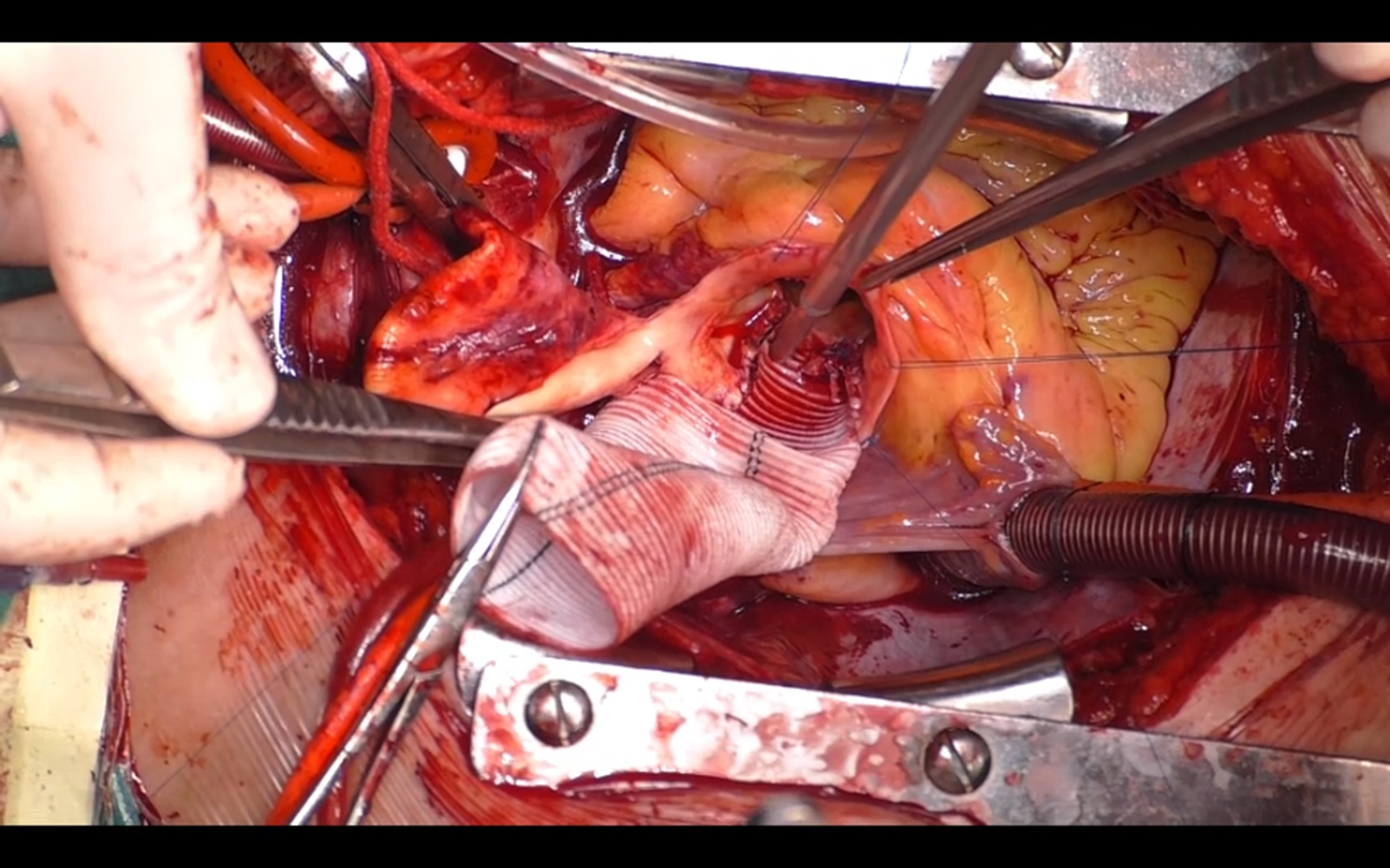

Supplement: Video 1 — A novel procedure that combines Y-incision aortic root enlargement with ascending aorta replacement. Video available at: https://www.jtcvs.org/article/S2666-2507(25)00339-6/fulltext. [file fx2.jpg]
